# Should the Increased Awareness of the One Health Approach Brought by the COVID-19 Pandemic Be Used to Further Tackle the Challenge of Antimicrobial Resistance?

**DOI:** 10.3390/antibiotics10040464

**Published:** 2021-04-20

**Authors:** Mohamed Rhouma, Michelle Tessier, Cécile Aenishaenslin, Pascal Sanders, Hélène Carabin

**Affiliations:** 1 Department of Pathology and Microbiology, Faculty of Veterinary Medicine, Université de Montréal, 3200 Sicotte, Saint-Hyacinthe, QC J2S 2M2, Canada; cecile.aenishaenslin@umontreal.ca (C.A.); helene.carabin@umontreal.ca (H.C.); 2 Groupe de Recherche en Épidémiologie des Zoonoses et Santé Publique, Université de Montréal, 3200 Sicotte, Saint-Hyacinthe, QC J2S 2M2, Canada; michelle.tessier@canada.ca; 3 Public Health Agency of Canada, 3200 Sicotte, Saint-Hyacinthe, QC J2S 2M2, Canada; 4 Centre de Recherche en Santé Publique de l’Université de Montréal et du CIUSSS du Centre-Sud-de-l’Île-de-Montréal, Montréal, Québec, QC H3N 1X9, Canada; 5 Laboratory of Fougères, French Agency for Food, Environmental and Occupational Health & Safety, ANSES, 35306 Fougères, France; pascal.sanders@anses.fr

**Keywords:** antimicrobial use, antimicrobial resistance, COVID-19, SARS-CoV-2, farm animals, one health

## Abstract

Several experts have expressed their concerns regarding the potential increase in antimicrobial resistance (AMR) during the COVID-19 pandemic as a consequence of the increase in antimicrobial and biocide use in humans globally. However, the impact of the pandemic on antimicrobial use (AMU) and AMR in animals has yet to be discussed and evaluated. Indeed, veterinary practices have been hugely impacted by the pandemic and its restrictive measures around the world. In this perspective, we call for more research to estimate the impact of COVID-19 on AMU and AMR in both humans and animals, as well as on the environment, in coherence with the One Health approach. In addition, we argue that the current pandemic is an opportunity to accelerate the implementation of a One Health approach to tackle the AMR crisis at the global scale. Indeed, the momentum created by the increased general awareness of both the public and decision-makers for the development and maintenance of effective drugs to treat human infections, as well as for the importance of a One Health approach to prevent the emergence of infectious diseases, should be used as a lever to implement global collaborative and sustainable solutions to the complex challenges of AMR.

## 1. Introduction 

When first identified in December 2019, in Wuhan (Hubei, China), coronavirus disease 2019 (COVID-19), caused by the severe acute respiratory syndrome coronavirus 2 (SARS-CoV-2), was described as a mystery viral pneumonia outbreak [1]. A few months after, the World Health Organization (WHO) declared a global pandemic on 11 March 2020. The uncertainties surrounding the severity, mortality, treatment, transmission dynamics, risk factors, individual and herd immunity of this new global threat led some to qualify it as one of “unknown unknowns” [2]. As of March 2021, there is still no solid evidence about the effectiveness of existing antivirals or other drugs for the treatment of COVID-19, nor is there any long term study about the duration of protective immunity after infection with SARS-CoV-2 or following vaccination against the disease [3]. Moreover, the recent emergence of SARS-CoV-2 variants, which could escape naturally in induced or vaccine-induced immunity, is a current global concern [4].

Multiple countries experienced multiple waves of cases and hospitalizations, pushing authorities to implement strict public health measures. This unprecedented global health issue is exerting a colossal pressure on scientists and physicians to develop and use therapeutic and prophylactic strategies to counter the severe consequences of this infection, so that societies can return to some form of normality and the economy can be restarted. Increasing evidence suggests that this context has resulted in an increase in both AMU and AMR in humans, followed by an increase in AMR bacteria shed into the environment [5,6,7]. It is noteworthy that AMR is a complex issue affecting the health of humans, animals and the environment on a global scale. Thereby, addressing this problem requires a coordinated multisectoral and multidisciplinary approach, such as the One Health approach [8,9]. This approach recognizes that human health and animal health are interconnected and linked to their shared environment [8]. According to the WHO, the areas of interest in which a One Health approach is particularly relevant include food safety and the control of both zoonoses and AMR [10].

In this perspective we: (i) give an overview of the trends in AMU during this pandemic and its consequences on humans and the environment; (ii) explore the potential consequences of the pandemic on AMU/AMR in veterinary medicine; and (iii) explain how the pandemic is an opportunity to strengthen the One Health approach for AMR prevention and control.

## 2. Impact of the COVID-19 Pandemic on Antimicrobial Use and Antimicrobial Resistance in Humans and in the Environment 

In May 2016, an independent review on AMR reported that AMR infections in humans were estimated to cause at least 700,000 deaths per year and this number is projected to increase up to 10 million deaths per year by 2050 globally, overtaking the number of people dying from cancer (8.2 million each year) [11]. In the United States, it is estimated that each year at least 2.8 million people get an antibiotic-resistant infection, and that more than 35,000 people die as a consequence of these resistant infections [12]. The Council of Canadian Academies estimated that AMR had caused 5400 human deaths (almost 15 human death per day) in Canada and cost the Canadian healthcare system nearly CAD 1.4 billion in 2018 [13]. On the other hand, many publications reported that the majority of hospitalized patients with COVID-19 were treated with antimicrobials, mostly to prevent or to treat secondary bacterial and fungal infections, despite a low proportion of these coinfections [14,15,16]. Indeed, Chen et al. have reported that around 71% of the hospitalized COVID-19 patients, admitted at Jinyintan Hospital in Wuhan (China) from 1 January to 20 January 2020, had received antimicrobial drugs (cephalosporins, quinolones, carbapenems, tigecycline, linezolid, and antifungal drugs), while only 1% and 4% demonstrated secondary bacterial or fungal coinfections, respectively [14]. Moreover, in a large multicenter Chinese study, 58% of patients admitted to hospitals as of 29 January 2020, received intravenous antimicrobials (without specification regarding the molecules used) [16]. Moreover, Wu et al. reported that, in three hospitals of Jiangsu Province, China (from 22 January to 14 February 2020), all patients were treated empirically with a single antibiotic, mainly moxifloxacin, for a period of 3 to 12 days (median 7 days) [15]. In addition, an international survey, performed in April 2020 amongst 166 participants from 23 countries and 82 different hospitals, suggested that broad-spectrum antimicrobials were commonly used in patients with COVID-19 [7]. Rawson et al. performed a review of the medical literature published between 1 January 2020, and 18 April 2020, to explore commonly reported bacterial/fungal coinfections in patients admitted to hospitals with lower respiratory tract infections associated with coronavirus [6]. The results of this review showed that the use of broad-spectrum antimicrobial therapy was widely reported, with 72% of COVID-19 cases receiving antibacterial therapy, while only 8% of the patients were reported as experiencing bacterial/fungal coinfections [6]. As such, prescribing of antimicrobials is significantly higher than the prevalence of bacterial/fungal coinfections, suggesting an empirical use of antimicrobials in patients with COVID-19, and such practice could increase the emergence of multidrug-resistant microorganisms as well as the development of other microbial infections (e.g., *Clostridioides difficile* infection) [17,18]. Consequently, in July 2020, the WHO warned of the risk of AMR spread as well as an impairment to antimicrobial stewardship as a result of the misuse of antimicrobials during this pandemic [19]. While some studies, mostly from Asia and the United States, have shown evidence about the negative impact of COVID-19 on AMR, other studies from France and Spain did not show an increase in infections with multidrug-resistant bacteria during the COVID-19 pandemic [20]. This conflicting evidence highlights that the impact of the pandemic on AMR in humans remains largely unknown, thereby more research is needed to better estimate the consequences of the unnecessary use, in just a few months, of high levels of antimicrobials on AMR development and spread worldwide.

In addition, considerable amounts of these antimicrobials could reach the environment in their active forms and might exert selection for resistant bacteria [21,22]. Moreover, the use of sanitizers and other biocidal agents (e.g., quaternary ammonium compounds (QACs), hydrogen peroxide, sodium hypochlorite, peroxyacetic acid, chlorine dioxide) has dramatically increased during the COVID-19 pandemic [23], hand hygiene and surface disinfection being among the most important preventative measures used globally to reduce SARS-CoV-2 transmission. Antimicrobial soaps and disinfectant cleaners could contaminate the environment, mostly through wastewater, in high concentrations and could select for AMR microorganisms [23,24,25]. Indeed, cross-resistance to clinically used antimicrobials has occurred following bacterial exposure and adaptation to some biocides [26]. It should be noted that quaternary ammonium compounds (QACs) constitute the highest percentage of biocidal agents in EPA approved disinfectant products for COVID-19 disinfection [27]. Many studies reported a cross-resistance to QACs and antimicrobials in *E. coli*, *Salmonella* and *Pseudomonas* strains [28,29]. In fact, a subinhibitory concentrations of some QACs (e.g., benzalkonium chloride (BAC), didecyl dimethyl ammonium chloride (DDAC)) can select for bacteria resistant to medically important antibiotics such as ampicillin, cefotaxime, ceftazidime, ciprofloxacin and colistin [28,29]. Moreover, it was reported that the exposure of *Pseudomonas aeruginosa* isolates to increasing concentrations of BAC selected for mutations in the polymyxin resistance (*pmrB*) gene, as well as for some physiological adaptations (including an overexpression of *mexCD-oprJ* multidrug efflux pump genes), contributing to a higher tolerance to polymyxin B and to other antimicrobials (e.g., ciprofloxacin, chloramphenicol, and rifampin) [30]. Common mechanisms such as bacterial membrane alterations and upregulation of efflux pumps are the most documented mechanisms responsible for bacterial cross-resistance to biocides and antimicrobials [26,31]. In addition, the use of these household cleaners, disinfectants, and sanitizers may be implemented globally for a prolonged time even beyond the COVID-19 pandemic, due to the change in world population’s behavior regarding hygiene, and such use could exert further selection for resistant bacteria to both antimicrobials and biocides.

## 3. Potential Impact of the COVID-19 Pandemic on Antimicrobial Use and Antimicrobial Resistance in Food-Producing Animals 

In farm animals, antimicrobials are used therapeutically (to treat clinically sick animals) for prophylaxis (to healthy animals at risk of infection), for metaphylaxis (to prevent infection among healthy animals in contact with infected animals), and some are still used for growth promoting purposes [9,32]. In 2010, the global antibiotic consumption in the livestock sector was estimated at 63,151 tons, with some models suggesting that it could increase by 67% to reach 105,596 tons by 2030 [33]. In the United States, antimicrobial use in farm animals was estimated to account for 80% of the nation’s annual antimicrobial consumption in 2010 [33]. The Public Health Agency of Canada estimated in 2018 that the livestock sector accounts for 79% of the total antimicrobial use in Canada [34]. It is noteworthy that of the 41 antimicrobials (including ionophores) that are approved for used in food-producing animals by the Food and Drug Administration (FDA), 31 are categorized as being medically important for human use [11]. While there is mounting evidence of a link between the preventative/growth promotion use of antimicrobials in animal production and the occurrence of AMR bacteria in humans and in animals [32,35,36], the relative contribution, in this issue, of AMU in farm animals remains unknown [37]. Despite this uncertainty, considerable efforts have been made in veterinary medicine to limit the spread of AMR bacteria and to preserve the effectiveness of antimicrobials [38,39,40]. Research on alternatives to the use of preventative antimicrobials in farm animals has increased in the recent years [41], especially following the ban of several classes of antimicrobials as growth promoters in several countries [38]. 

The impact of the COVID-19 pandemic on the global efforts to control AMU and AMR in animals is unknown. The following factors could have contributed to an increase in AMU and ultimately an increase in AMR. First, veterinary services were not considered essential in several countries implementing lockdowns, at least during the first wave of the pandemic, and consequently veterinarians were not able to offer preventative care, including vaccination, to their clients (farmers, pet owners, etc.). This could have resulted in animals developing infections which would normally be prevented and hence an increase in AMU in animal production. Second, the unavailability of veterinary services, particularly during the first lockdown, could increase self-medication as well as off-label use of antimicrobials on farms. Furthermore, the pandemic has also resulted in instances where animals were kept on farms for longer than usual due to large outbreaks in slaughterhouses and the disruption of inter-regional and international transportation. This situation might lead to an increase in AMU in animal production as a consequence of the increase in animal density on farms, which could facilitate the spread of infectious diseases. In addition, all research and development activities of alternatives to antimicrobials and vaccines in animals could be disrupted worldwide during this pandemic, which could create more pressure on the AMU in animals in the long term. 

On the other hand, the pandemic may have also resulted in a decrease in AMU in farm animals. Indeed, access to antimicrobials (or their molecules or ingredients) could have been disrupted by commerce and transport limitations. Moreover, the pandemic may have caused a breach in surveillance for AMU and AMR in food systems globally. For example, in several countries (Canada and France, for example), the majority of animal health laboratories were redirected towards COVID-19 diagnoses in support of the public health laboratories. Food safety regulatory agencies also faced a huge challenge related to staff shortages, resulting in a reorientation of the inspection and the sampling activities towards certain sectors (e.g., meat processing plants), rather than sampling for AMR monitoring, to avoid further overloading of the human health systems with foodborne infections. Sampling along the food chain (on farms, at slaughterhouses, and from retail meats) and the analysis for the surveillance of AMR for some foodborne pathogenic bacteria (e.g., *Salmonella*, *Campylobacter*, *E. coli*) were greatly altered by the pandemic and particularly during the first lockdown. The extent to which the pandemic led to an increase or a decrease in AMU and ultimately its impact on AMR in animal populations should be urgently assessed in future studies.

## 4. Lessons Learned from the COVID-19 Pandemic to Strengthen the One Health Approach for the Control of Antimicrobial Resistance

By the end of February 2021, more than 100 million people had been diagnosed with SARS-CoV-2 and 2.5 million people had died of the disease it causes globally [42]. The ongoing pandemic constantly reminds the public of the importance of hand-washing and there has been a growing interest in vaccination against influenza and pneumococcal disease, which, in combination with the current efforts to social distance and wear masks, will likely decrease the spread of other airborne infections [43]. Moreover, to prevent COVID-19 contamination, several interesting communication tools (e.g., brochures, flyers, advertising spot) have been developed by health authorities around the world in order to educate, while simplifying the information, all citizens, even those in the most remote regions of the planet, so they adhere to some precautionary COVID-19 measures (e.g., wearing a face mask, social distancing). This has been accompanied by important media coverage, investigations and interviews with experts regarding emerging infections, zoonoses, public health and the One Health approach. Such communication tools are also very relevant to raise awareness regarding AMU and AMR at the human–animal–environment interface. In fact, awareness campaigns on AMR through effective education and communication constitute the first goal of the WHO Global Action Plan on Antimicrobial Resistance [44]. In the current context, all humanity is very aware of the importance of having effective drugs to treat microbial infections and also the necessity to protect their effectiveness through time. This aspect is much more important in the current context of AMR, where multidrug-resistant bacteria have spread widely and the number of brand new drugs placed on the market is drastically decreasing. Indeed, the last new class of antimicrobial discovered is daptomycin (1986), which was only approved in 2003 by the US Food and Drug Administration (FDA), showing that antimicrobial agents found on the market in the last 30 years are associations or redevelopments of classic antimicrobials [45]. Thereby, it is the responsibility of all stakeholders involved in AMU (e.g., physicians, pharmacists, patients, veterinarians, agronomists, farmers and regulatory agencies) to develop various strategies ensuring the responsible use of antimicrobials in order to protect their effectiveness for as long as possible. 

Environmental changes and ecosystem degradation across the planet (e.g., deforestation, intensified agriculture and livestock production, illegal and poorly regulated wildlife trade) increased the frequency of contacts between wild animals, domestic animals and humans, contributing to a zoonotic transfer of diseases [46]. Thereby, the COVID-19 pandemic is bringing to light the importance of protecting the environment and the ecosystem, which is of paramount importance in the context of AMR management. Indeed, there is growing evidence that the environment plays an important role in the transmission of AMR bacteria and/or AMR genes to humans and livestock in addition to serving as a reservoir of AMR microorganisms. For example, *Shewanella algae*, an environmental species from marine and fresh water, was identified as a reservoir of plasmid-mediated quinolone resistance (QnrA) in *Enterobacteriaceae* [47]. Cabello et al. suggested that mobile colistin resistance (*mcr*) genes may have originated in aquatic environments as a result of aquaculture activities, and these genes could have earned terrestrial bacteria by horizontal gene transfer to yield colistin-resistant bacteria in humans and animals [48]. It should be stressed here that the United Nations Environment Programme (UNEP) ranked environmental AMR first among the six emerging issues of concern (environmental dimensions of AMR, nanomaterials, protected marine areas, sand and dust storms, off-grid solar solutions, and environmental displacement) [49]. It is therefore essential to establish science-based standards regarding the acceptable antimicrobial concentrations (and AMR genes) in soil (manured or not), in aquaculture, in farm and hospital environments as well as in manufacturing effluents in order to better inform and involve policy-makers in the management of this issue [50]. 

Finally, by joining the few episodes of infectious diseases that have deeply shaped human history, the COVID-19 pandemic had succeeded, in an unprecedented way, in increasing awareness of both the public and decision-makers regarding the development and maintenance of effective drugs to treat human infections as well as for the importance of the One Health approach to prevent the emergence of infectious diseases. We believe that these prerequisites should be used as a lever to accelerate both the development of global collaborations and the implementation of sustainable solutions for the management of the current AMR crisis.

## 5. Conclusions

Antimicrobial resistance is a cross-sectoral complex problem affecting the health of humans, animals and the environment. We strongly believe that COVID-19 pandemic has generated a powerful incentive and momentum to address the AMR crisis by accelerating collaborations and interdisciplinary communication between concerned stakeholders (e.g., researchers, physicians, veterinarians, pharmacists, farmers, other health and environmental professionals, public and policy-makers), while taking into account the specificity of each sector during and beyond this pandemic. More research and retrospective analysis of surveillance data are needed in both humans and animals as well as in the environment to estimate the impact of COVID-19 on AMU and AMR in coherence with the One Health approach. One of the lessons of the COVID-19 pandemic is that acting too late carries serious costs to both health and the economy. Thereby, the colossal budgets allocated to human medicine worldwide for the control of this pandemic should not compromise efforts conducted to manage the current AMR crisis at the human–animal–environment interface. We stress here the paramount importance of the One Health approach to face the increasing threat of AMR, and we believe that this pandemic could be an excellent opportunity to accelerate its implementation for an effective surveillance, prevention and control of AMR at the global scale.
